# High throughput quantitative proteomic analysis of cellular host response to influenza virus in primary human monocyte-derived macrophages

**Published:** 2011-01-10

**Authors:** Chung Yan Cheung, Eric Chan, Carolina Ka Long Leung, Jon Jacobs, Richard Smith, Michael Katze, Malik Peiris

**Affiliations:** 1 Department of Microbiology, Li Ka Shing Faculty of Medicine, The University of Hong Kong, Queen Mary Hospital, Hong Kong SAR; 2 Department of Microbiology, University of Washington, Seattle, WA, USA; 3 Washington National Primate Research Center, University of Washington, Seattle, WA, USA; 4 Environmental Molecular Sciences Laboratory, Pacific Northwest National Laboratory, Richland, WA, USA; 5 HKU-Pasteur Research Centre, Hong Kong, Hong Kong SAR

## 

Host response to infection with pathogens such as influenza viruses serves to primarily generate immune responses in an attempt to counter the invading pathogens. Consequently, how the host interacts with the virus not only has important influence on its pathogenesis, but also its transmission in a population and dissemination within the infected host. On the other hand, an overly active immune response may actually lead to excessive inflammation, which could be detrimental to the host as many lines of evidence have suggested for human H5N1 infection and the 1918 H1N1 pandemic virus. Macrophages are key orchestrator of the immune response and being one of the most abundant cell types in the respiratory system that can be infected with influenza viruses. Therefore, this study aims utilize high-throughput mass spectrometry to compare time series global proteomic profiles of primary human monocyte-derived macrophages infected with highly pathogenic H5N1 and seasonal H1N1 influenza viruses, in order to allow combinational analysis of proteomic datasets with existing body of transcriptomic datasets to provide further insight into the cellular and molecular host response to influenza virus infection. Global proteomic profiling of influenza virus infected macrophages revealed that many of the proteome changes could not be accounted for by transcriptomic profiling with microarrays. The low concordance of the global proteome profiling results with transcriptomic data indicates that high throughput quantitative proteomic analysis can provide a significant additional dimension to enable combinational analysis with existing datasets from studies using high-throughput genomic platforms. Results from this study suggest proteome changes in infected macrophages that were common to both viruses could be involved in processes that are similar, such as viral replication as both viruses replicate equally well in our macrophage model. On the other hand, pathways derived from analysis of differentially affected proteomes may contribute to the high pathogenic nature of H5N1 viruses. Therefore systematic integrative analysis of datasets from different sources could significantly contribute to detecting differentially expressed genes or pathways that are of relevance during virus infection and allows assessment of heterogeneity. This is likely to contribute to target identification for host-directed treatment of disease caused by influenza viruses.

